# Environmentally-Controlled Near Infrared Spectroscopic Imaging of Bone Water

**DOI:** 10.1038/s41598-019-45897-3

**Published:** 2019-07-15

**Authors:** Ramyasri Ailavajhala, Jack Oswald, Chamith S. Rajapakse, Nancy Pleshko

**Affiliations:** 1 Department of Bioengineering, Temple University, Philadelphia, USA; 2 Departments of Radiology and Orthopaedic Surgery, University of Pennsylvania, Pennsylvania, USA

**Keywords:** Engineering, Biomedical engineering, Engineering, Biomedical engineering

## Abstract

We have designed an environmentally-controlled chamber for near infrared spectroscopic imaging (NIRSI) to monitor changes in cortical bone water content, an emerging biomarker related to bone quality assessment. The chamber is required to ensure repeatable spectroscopic measurements of tissues without the influence of atmospheric moisture. A calibration curve to predict gravimetric water content from human cadaveric cortical bone was created using NIRSI data obtained at six different lyophilization time points. Partial least squares (PLS) models successfully predicted bone water content that ranged from 0–10% (R = 0.96, p < 0.05, root mean square error of prediction (RMSEP) = 7.39%), as well as in the physiologic range of 4–10% of wet tissue weight (R = 0.87, p < 0.05, RMSEP = 14.5%). Similar results were obtained with univariate and bivariate regression models for prediction of water in the 0–10% range. Further, we identified two new NIR bone absorbances, at 6560 cm^−1^ and 6688 cm^−1^, associated with water and collagen respectively. Such data will be useful in pre-clinical studies that investigate changes in bone quality with disease, aging and with therapeutic use.

## Introduction

Water is an important contributor to bone quality, and makes up ~approximately 10% of cortical bone wet weight^1^. Increased fracture risk and bone fragility have been associated with a decrease in the overall skeletal tissue water content^2–4^. However, specific regions associated with water changes have not been fully elucidated. In cortical bone, water generally can be found as tightly and loosely (surface) bound to either collagen and mineral, and within the pore network. In collagen, the helical structure is stabilized via intramolecular and intermolecular hydrogen bonding of water molecules^5^. In mineral, water molecules are in part tightly bound to carbonated crystals in the apatite core^6^. Water is also loosely bound to the amino acid side chains in collagen, as well as to the surface ions in mineral crystals. Additionally, surface water is also found between the collagen and mineral interphase^1,7^.

Cortical bone water content has been evaluated through several destructive and nondestructive techniques^1,2,8–11^. Gravimetric analysis is considered a gold standard method for evaluation of water content in biological tissues^12^. Nevertheless, since this method is destructive and time consuming, it has motivated development of nondestructive techniques that can be applied for assessment of water related to bone quality. One such method is NIRSI, a technique based on radiation in the near infrared region (NIR) of the electromagnetic spectrum, 4000–13000 cm^−1^. The sensitivity of NIR to water has been widely studied as a quality parameter in the food science, agricultural and pharmaceutical industries^13–18^. NIR spectra are comprised of overtones and combination absorbance bands from molecular vibrations of C-H, O-H, S-H and N-H bonds^19,20^. However, a typical spectrum in the NIR consists of overlapping bands dominated by water O-H absorbances, which can make it challenging to interpret spectral absorbances from non-aqueous components. In contrast, one primary advantage of NIR is that it penetrates several millimeters into a sample, which permits a full-depth chemical analysis of biological tissues and materials^21^.

NIRSI has been utilized to study water content in musculoskeletal tissues such as cartilage and bone. Padalkar *et al*.^22^ evaluated water content in articular cartilage by gravimetric and NIR techniques, and NIRSI helped to differentiate between free (6890 cm^−1^) and bound (5200 cm^−1^) water in cartilage. More recently, Rajapakse *et al*.^20^ non-destructively evaluated compositional changes in aging cadaveric cortical bone tissues by NIRSI for skeletal quality assessment, and correlated matrix (4608 cm^−1^) and water absorbances to MRI-derived water content in the same tissues. Together, both studies showed that NIRSI is a sensitive method for evaluation of changes in tissue water content, and correlation to other conventional techniques.

Other nondestructive techniques that have been used to evaluate cortical bone water content are Raman spectroscopy^1,11^ and magnetic resonance imaging (MRI)^20,23^. Unal *et al*.^1^ conducted dehydration studies in bovine cortical bone samples to determine Raman spectral water absorbances that can be used as biomarkers for bone quality assessment. That study labeled Raman spectral intensities for water compartments in cortical bone as bulk water, collagen-bound water, mineral-bound water and water associated with mineral and collagen^24^. Even though individual peak intensities at specific time points of dehydration where useful for assessment of changes in water, the instrumentation used for the analyses was custom built, and not widely available. Many common Raman spectrometers produce spectra that can have very low signal to noise ratios, and fluorescence interference can be a problem during data analysis^25^. MRI imaging of water has the advantage of being applicable to clinical studies, and several studies have investigated changes in water content in tissues for bone quality assessment^23,26,27^. Drawbacks of MRI analyses are that only collagen-bound water and pore water can be detected currently, and mineral-bound water signatures are not available. Further, in clinical MRI studies, the best resolution is generally on the order of a few hundred microns. In contrast, NIRSI will allow for data acquisition at a pixel resolution as high as 6.25 microns^20,28^.

One major challenge in evaluation of water content using spectroscopic techniques is the constant fluctuations in hydration and dehydration of tissues during data collection. Recent studies that have evaluated cortical bone water content using NIRSI and Raman spectroscopy have acquired data in atmospheric conditions^1,20^. Depending on the atmospheric humidity, and the time required for data collection, tissues may undergo a reoccurring change in their water content which may be reflected over time in their spectra by a potential increase or decrease at the frequencies of water absorbances. Consequently, the repeatability of studies may be challenging, in particular during water calibration studies^29^.

Motivated by the challenges with water vapor fluctuations, and the need to definitively identify NIR absorbances from mineral and collagen components in bone, we designed and implemented an environmentally-controlled chamber to optimize the collection of NIR spectral imaging data from biological tissues. This is a partially sealed chamber that allows for air flow at a constant humidity level. Thus, the water loss and gain during data collection is reduced and changes in atmospheric factors, such as water absorbances, are minimized. Here, we describe the chamber, and subsequent experimental NIRSI data obtained from serially dehydrated bones with a range of water content. Utilizing multivariate analysis, we established a calibration curve from which gravimetric water content can be predicted from NIR spectral data obtained from human cortical bone in the chamber. In addition, serial dehydration of bone enabled identification of absorbances attributed to non-aqueous components of bone. These studies will provide a firm foundation for NIRSI evaluation of water content in harvested bone, and for pre-clinical studies of bone quality.

## Results

**Figure 1:**
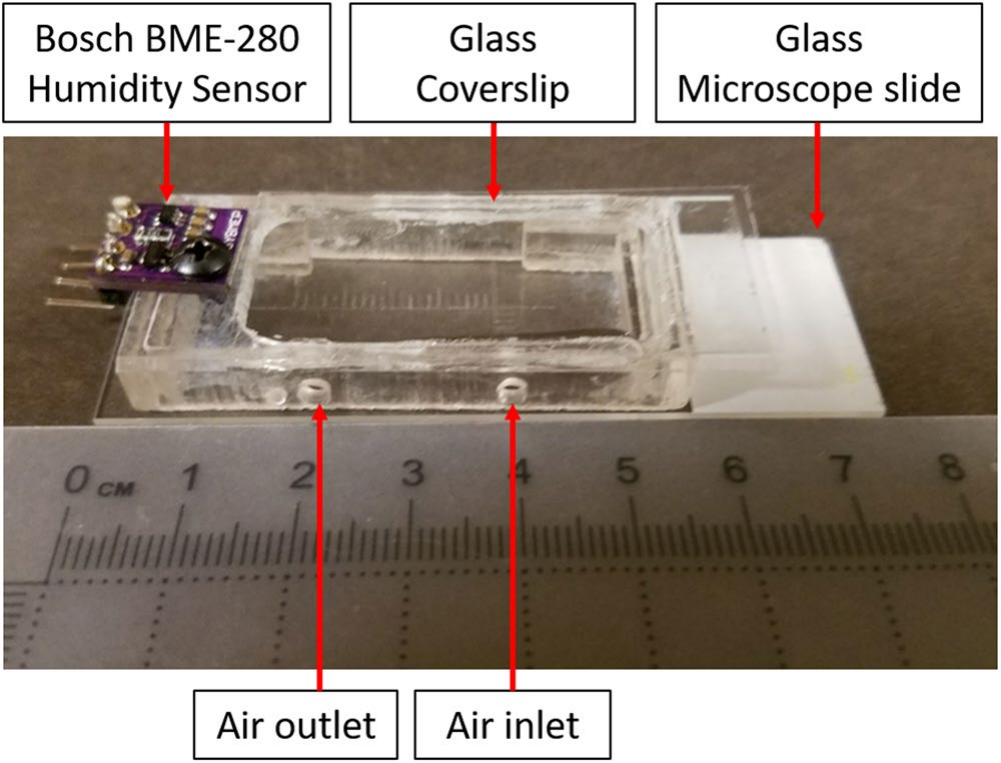
Small environmental chamber with input and output for airflow tubing, and humidity sensor.

**Figure 2:**
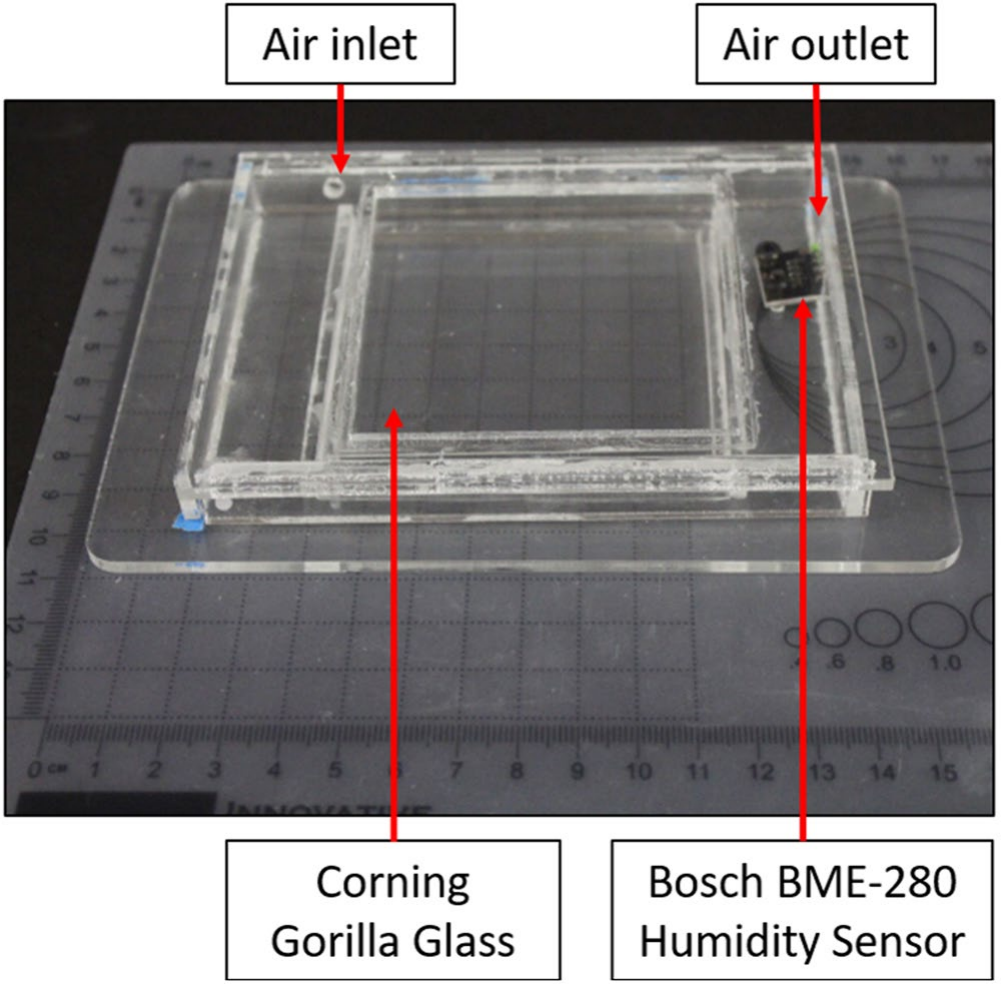
Large environmental chamber with input and output for airflow tubing, and humidity sensor.

The results described below are based on the following approach where we assessed the optimal sampling method using environmental chambers. Initially, a small chamber was designed to evaluate NIR data collection from wet (hydrated) and dry bone samples in low and high relative humidity (RH), with the goal of determining which environment was most stable for data collection. Since the imaging area in the small chamber was limited by the size of the cover slip (Fig. 1), a large chamber was subsequently designed and validated for data collection from hydrated and dry clinical size bone samples (described following the small chamber results) (Fig. 2).

### Optimization of spectral data collection in an environmentally-controlled chamber

**Figure 3:**
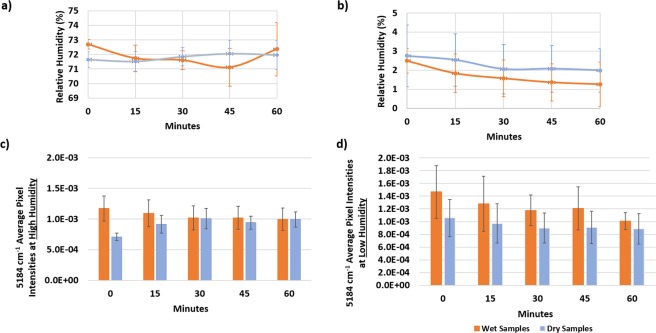
RH inside the imaging chamber during one hour of spectral data collection from wet and dry bone samples in high (**a**) and low (**b**) RH conditions. Average pixel intensity at 5184 cm^−1^ (inverted second derivative intensity of NIRSI water absorbance) inside the imaging chamber during one hour of spectral data collection from wet and dry bone samples in high (**c**) and low (**d**) humidity.

The small chamber (Fig. 3) continuously maintained either high or low RH for 60 minutes during NIRSI data collection (Fig. 3a,b). Collection of spectral imaging data from dry bone in a humidified environment resulted in absorption of moisture and a higher water content after 15 minutes of data collection (Fig. 3c). However, spectral acquisition from hydrated bone in a humidified environment resulted in minimal water absorption, likely due to those samples already being saturated (Fig. 3c).

Conversely, in a low RH environment, neither hydrated or dry bone absorbed or lost a significant amount of water, and the water absorbance from the bone samples remained stable (Fig. 3d). Accordingly, low RH was determined the optimal condition for creation of the calibration curve from serially dehydrated bone samples, and the large chamber (Fig. 2) was only validated with low RH.

The validation of the large chamber was performed by periodic NIRSI data collection from marked areas of bone samples, combined with evaluation of changes in a well-defined NIR bone water absorbance at 5184 cm^−1^. Once the data collection parameters were optimized, a NIRSI water calibration curve from cadaveric human bone samples of uniform thickness was developed using multivariate spectroscopic methods. Further, NIR absorbances from non-aqueous components of bone were elucidated by analysis of dehydrated bone tissues.

**Figure 4:**
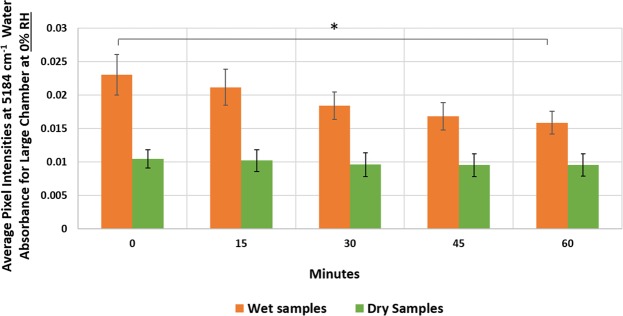
Average NIRSI pixel intensities at 5184 cm^−1^ (water content) of wet and lyophilized bone samples under 0% RH in the large chamber. Wet samples gradually lose water content over an hour, while dry samples maintain their water content. (*) Average pixel intensities at 5184 cm^−1^ for wet sample group at 0 and 60 minutes were statistically significant at p < 0.05.

The large chamber successfully maintained 0% relative RH for 60 minutes of data collection (see Supplementary Fig. S1). With NIRSI data collection from fully hydrated bone samples, an initial drop in intensity of the 5184 cm^−1^ water absorbance from 0 minutes to 15 minutes was observed, believed to be attributable to remnants of PBS evaporating from the surface of the bone (Fig. 4). The water content in hydrated bone incrementally decreased during 60 minutes of NIRSI imaging in low RH resulting in ~ 31% water loss in bone overall (Fig. 4), with a statistically significant reduction in water content between the 0- and 60-minutes time points. However, for the dry bone samples, the average pixel intensity at 5184 cm^−1^ did not significantly change with time, and the chamber was able to maintain low constant RH for 60 minutes of imaging (Fig. 4). Based on these results, it was determined that calibration curve data collection, which would span collection of data from both hydrated and dry samples, could occur in low constant RH. However, data collection should start after a short drying period to minimize changes from bulk surface water loss and should not exceed 5 minutes. This would ensure that no significant changes in water content of the more hydrated samples would occur.

### Identification of absorbances from non-aqueous components of bone from dehydration studies

**Figure 5:**
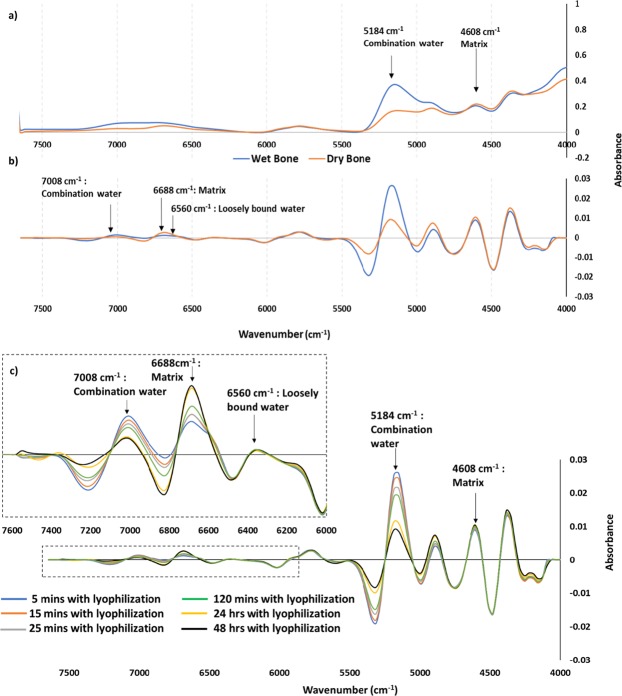
(**a**) Raw NIR spectra of wet (hydrated) and dry bone. (**b**) NIR second derivative of wet (hydrated) and dry bone. The water and matrix peaks are more resolved in the second derivative spectra compared to raw spectra. A reduction in the 5184 cm^−1^ water peak can be seen in both raw and second derivative spectra of dry bone. (**c**) Second derivative NIR spectra of serially dehydrated bone. The absorbance of the water peaks (5184 cm^−1^, 6560 cm^−1^ and 7008 cm^−1^) decreased with increasing lyophilization time.

Raw (Fig. 5a) and second derivative spectra (Fig. 5b) obtained from serially-dehydrated samples were used for evaluation of changes in NIR bone water peak intensities at 5184 cm^−1^, 6560 cm^−1^ and 7008 cm^−1^, and to elucidate NIR absorbances related to non-aqueous cortical bone components. The two main NIR bone matrix (collagen) absorbances were observed at 4608 cm^−1^, a combination peak from C-H stretches and at C-H deformation, and at 6688 cm^−1^, a 1^st^ overtone absorbance from N-H stretching from amino acid side chains of collagen^19^. Mineral-associated absorbances in cortical bone still need to be further validated, but based on a recent study, a potential P-OH vibration in the NIR range can be observed at approximately at 7000 cm^−1^ ^30^.

Comparison of spectra from wet and dry shows that with serial lyophilization, there was a reduction in water absorbances at 5184 cm^−1^ and 7008 cm^−1^ but at 48 hours, features in those regions were still present; in contrast, only a negligible absorbance was observed at the 6560 cm^−1^ water absorbance. Therefore, the 6560 cm^−1^ water absorbance is suggested to arise from loosely bound water that can be fully removed with lyophilization, whereas the water absorbances at 5184 cm^−1^ and 7008 cm^−1^ have loosely and tightly bound water components, since the peaks reduce in intensity, but are still present with increasing lyophilization time. Thus, all three water absorbances have a loosely bound component, and two have both loosely and tightly bound water components. Another possible interpretation is that there are matrix absorbances that underlie the water absorbances at 5184 cm^−1^ and 7008 cm^−1^, and thus all three of the major water absorbances may be “loosely bound” only. Further studies involving deuterium exchange would enable a conclusive determination of this. Interestingly, the 6688 cm^−1^ matrix peak becomes increasingly significant as the 6560 cm^−1^ loosely bound water diminishes (Fig. 5c).

#### Creation of a water calibration curve using an optimal spectral data collection method

Dehydration of bone through lyophilization affected the water and matrix NIR absorbances. Univariate correlation analysis between gravimetric water content either alone, or ratioed to a matrix peak, and NIR water absorbances, 5184 (R = 0.84, p < 0.05), 7008 (R = 0.63, p < 0.05), 5184/4608 (R = 0.96, p < 0.05) and 7008/6688 (R = 0.90, p < 0.05) showed a significant linear correlation between the two variables (see Supplementary Table 1). Additionally, multiple liner regression (MLR) models were developed to predict gravimetric water content using individual water and matrix absorbances. In both the MLR models, the root mean square error (RMSE) for prediction based on intensities of the second derivative peaks at 5184 + 4608 (R = 0.95, p < 0.05, RMSE = 0.67, 8.90%) and 7008 + 6688 (R = 0.93, p < 0.05, RMSE = 9.30%) were comparable (See Supplementary Table 1).

**Figure 6:**
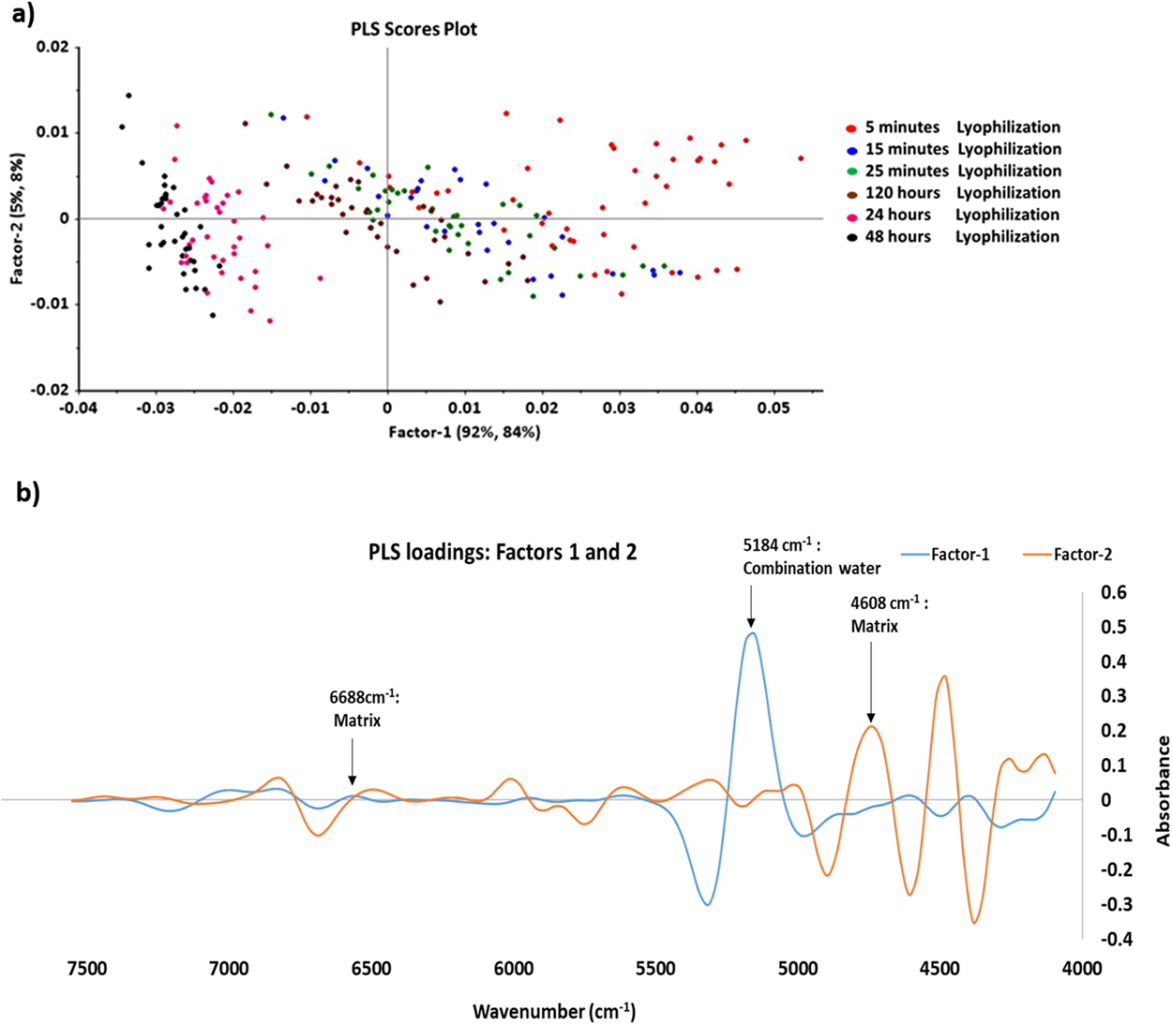
(**a**) The lyophilization time points separate from right to left, reflective of increasing lyophilization time. (**b**) Factor 1, which underlies most of the data separation, is dominated by the 5184 cm^−1^ water absorbance, while factor 2 is dominated by matrix absorbances.

**Figure 7:**
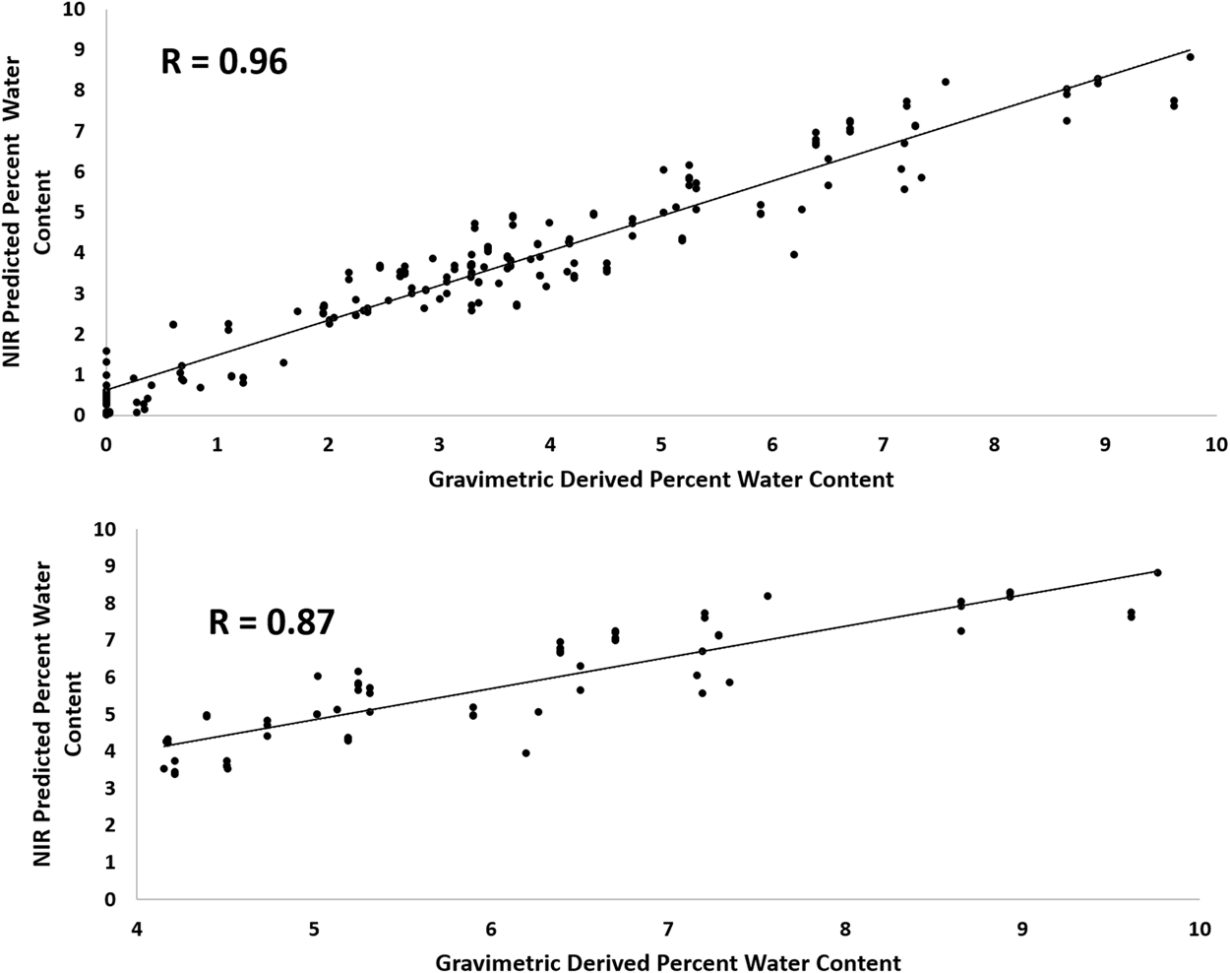
Independent NIRSI prediction of gravimetric water in cortical bone over the (**a**) 0–10%, and (**b**) 4–10% (physiologic) water content ranges. RMSEP for the two models 0–10% range and 4–10% range are 7.39% and 14.5% respectively.

PLS models were developed to correlate NIR spectral data to gravimetrically-determined water content. As seen in the scores plot (Fig. 6a), 92% of the variance among samples was primarily attributable to the changes in the water absorbance at 5184 cm^−1^ in factor 1 of the loadings plot. Additionally, factor 2 was dominated by matrix peaks, which influenced ~5% of the variance in the data (Fig. 6b). Three independent prediction models were developed that predicted water content with an average RMSEP of 7.39% of total water content. A significant correlation was seen between gravimetric and NIR-predicted water content in both the overall range of 0–10% wet weight (R = 0.96, p < 0.05, RMSEP = 0.67, 7.39%) (Fig. 7a) and in the physiologic range of 4–10% (R = 0.87, p < 0.05, RMSEP = 0.81,14.5%) (Fig. 7b). Interestingly, the RMSECV value (6.60%) for the best PLS model (see Supplementary Table 2) was lower but in a similar range as the errors of the MLR models (5184 cm^−1^ + 4608 cm^−1^ = 8.90%, 7008 cm^−1^ + 6688 cm^−1^ = 9.30%).

## Discussion

The current study demonstrates optimal conditions for collection of NIRSI data for evaluation of water from bone samples. Such methodology will be helpful for evaluation of changes in water in harvested pre-clinical samples from aging studies, and bone quality changes in therapeutic protocols. Clinically, bone mineral density (BMD) is considered the conventional technique for assessment of bone quality, which is related to bone strength, fracture risk and fragility^31,32^. However, BMD alone is not the sole indicator for fracture risk assessment. For example, changes in bone microarchitecture, cellular density, tissue organization, and compositional changes related to mineral and collagen water interactions can play a role in bone quality^33,34^. A recent study showed that BMD as an indicator accurately predicted only 50% of fractures^35^, and it had only a weak correlation to bone strength. This motivates researchers to investigate additional biomarkers that can be used as indicators for bone quality assessment^31,32,36,37^.

Compositional changes in cortical bone after the age of 30 result in a gradual decrease in bone mass and an increase in bone fragility^33,38–40^. Aging can trigger the displacement of collagen-associated water molecules due to increased glycation of crosslinking in collagen fibers^20^. Additionally, mineralization tends to decrease with age due to imbalances in the turnover process between bone resorption and formation^33^. Continuous bone loss with age leads to osteoporosis which is caused by a significant decrease in bone mass and an increase in bone porosity^40^. These compositional changes also decrease the structural integrity of cortical bone^11,41,42^. Nyman *et al*.^10,43,44^ performed extensive mechanical studies on bovine cortical bone and have shown that dehydration significantly decreases toughness, while stiffness increases^43,44^. Collagen provides the toughness in cortical bone via its inter and intra molecular bonding to water molecules; These studies indicate that in dehydrated bone, longitudinal contraction increases for collagen and the fibrils become stiffer leading to a greater risk of bone fracture^10^.

Anti-resorptive drugs used to treat osteoporosis such as Raloxifene act by binding to estrogen molecules to slow down the bone resorption process^45^. Gallant *et al*.^32^ have shown that Raloxifene-treated beagles had a 17% percent increase in cortical bone water without significantly altering tissue BMD levels. They also found that Raloxifene had a positive effect on increasing bound water at the collagen/mineral interphase which effectively caused an increase in the overall bone toughness. This study along with several others emphasize that the hydration of bone correlates with bone mechanics better than BMD levels^46–48^. Together, these studies concluded that toughness significantly decreases with age and with dehydrating bone. Therefore, there is a strong possibility that bone water content can be used as an indicator for bone quality assessment.

Several studies have investigated cortical bone water through MRI and spectroscopic techniques for assessment of bone compositional integrity^1,9,11,47,49–51^. Rajapakse *et al*.^20^ have previously suggested that 60% of the water content detected in cadaveric samples by UTE-MRI is associated with collagen bound water, while the remainder is pore-associated water. Additionally, studies in bovine cortical bone have also determined that 73% of water that is detected is collagen bound^47^. Chen *et al*.^49^ showed that the ratio of free to bound water increases with the progression of osteoporosis, Allen *et al*. through UTE-MRI analysis found that Raloxifene treated beagles had 14% more bound water compared to control samples^45^. Clearly, changes in bone water can provide insight into disease progression and therapeutic effectiveness in bone-related diseases.

Unal *et al*.^1^ also concluded that Raman spectroscopy is a feasible technique for determination of water content in tissues. In their study, they performed sequential dehydration experiments on bovine cortical bone and assigned water compartments for bone based on changes in Raman sub-bands under the broad OH-stretch. The data collection of dehydrating tissues took place in atmospheric conditions, with data collection time limited to 10 seconds per sample. While this method is feasible for wet samples, atmospheric water tends to infiltrate pores in lyophilized bone and may interfere with data collection and interpretation. In a recent study from our lab, we initially collected NIRSI data from 500-micron thick bone samples between two glass slides to minimize water loss during data collection^20^. However, atmospheric conditions could potentially interfere with data interpretation of serial dehydration and water calibration spectral imaging studies.

Accordingly, one main goal of the current study is to describe the importance of utilizing an environmentally-controlled chamber to assess water in cortical bone with NIRSI. NIR spectra are dominated by two previously reported water absorbances at 5184 cm^−1^ and 7008 cm^−1^,and one newly identified water peak at 6560 cm^−1^ ^20^. The slow dehydration of bone over time helped to elucidate an absorbance at 6688 cm^−1^ that arises from N-H stretching in matrix molecules, along with the 4608 cm^−1^ absorbance that was previously established as a matrix absorbance^19^. Serial lyophilization of bone aided in classification of NIR water absorbances as either loosely or tightly bound. Data collection in atmospheric settings affects the repeatability of studies as atmospheric moisture can accumulate on the surface of sample, or conversely, if in a dry environment, can result in water evaporation during data collection. The sensitivity of NIR spectra to water is well known, and it was essential to collect data in an environmentally-controlled chamber that has a constant flow of low humidity for reliable measurements of compositional components without the interference of external water for dehydrated samples. However, in the future, if NIR data are being collected from hydrated samples, one can use either a chamber with high RH, or a chamber with low RH humidity, with the low RH data collection contingent on a short data collection time.

Analysis of second derivative peak heights of water and matrix showed the first significant reduction in water absorbance occurred after 120 minutes of lyophilization (Fig. 5b). Since the 6560 cm^−1^ peak becomes insignificant with 48 hours of dehydration, it is suggested that after this timepoint any remaining water absorbances reflect tightly bound species. Additionally, since NIR spectra are dominated by water absorbances, the matrix peaks are better resolved with increasing dehydration time of the sample. For example, as the intensity of the 6560 cm^−1^ and 7008 cm^−1^ water peaks reduce, the adjacent matrix peak at 6688 cm^−1^ increases (Fig. 5b). Future studies will investigate the exact nature of the location of tightly bound water.

Both univariate and multivariate analysis were used to evaluate the spectral data. NIR inverted second derivative peak heights at 5184 cm^−1^ and 7008 cm^−1^ significantly correlated with gravimetrically-derived water content (see Supplementary Table 1). Additionally, MLR models successfully predicted gravimetric water content by using inverted second derivative peak height values of water and matrix absorbances.

PLS models showed strong correlations (R = 0.92) between NIR spectra and gravimetrically-derived percent water content. Factor 1 in the loadings plot clearly shows that the variation in the spectra related to water content arises from the water absorbances. Interestingly, factor 2 shows that after accounting for the water components, the matrix peaks influence the NIR spectral data as well. The correlation between spectral and gravimetric data in the physiologic range of 4–10% was somewhat lower (R = 0.86) compared to the full range data, in large part due to greater variation in the more hydrated tissues. One possible explanation is that residual surface water could still be evaporating during gravimetric data collection. Additionally, the rate of evaporation among the samples could have varied since some samples had larger surface areas compared to others, dependent on the exact porosity and area of the tissue.

As seen in Supplementary Tables S1 and S2, the errors obtained from the MLR and PLS models are comparable. Therefore, similar results can be obtained by using both techniques to quantity spectral data. However, the advantage of using PLS regression, as opposed to single or two frequency NIR correlations, is that correlations between spectral and gravimetric data can be found across the entire range of spectral frequencies. Thus, additional frequencies that contribute to prediction of the outcome data could be identified. Additionally, PLS describes the data by grouping samples based on similar characteristics (chemical and physical). As seen in Supplementary Tables S1 and S2, multivariate analysis error is lower than that from single peak analysis. Additionally, the loading plots in PLS models help to explain the variance that is seen in the entire data set. As observed in factor 1 and 2, the variation in the data set could be attributed to both changes in water and matrix absorbances at several frequencies.

As previously reported, the sensitivity of NIR to PO_4_ absorbances is very low compared to what is observed in the mid-infrared region, and thus the mineral phosphate component cannot be identified by these combination or overtone vibrations^30^. However, P-OH overtone vibrations originating from mineral crystals in synthetic and biological powders have been assigned to ~7000 cm^−1^ ^30^, which could be useful for mineral assessment in bone. These findings need to be further validated to accurately identify peak positions, in particular intact bone tissues. It will be important to differentiate the OH vibrations originating from the apatite core from OH vibrations from water molecules bound to the mineral.

Although the environmental chamber provided a stable environment in which to collect NIR data, this can also be considered a limitation of such studies. If this non-destructive technique for water assessment is to be truly useful in pre-clinical, or eventually clinical studies, it will be necessary to develop a method for data collection in atmospheric conditions. This would have to involve data collection in a limited time frame, to significantly reduce loss of water to the atmosphere, and may involve development or application of new instrumentation. Another limitation in transfer of this protocol to other samples is that here, all data collections were done using one spectrometer on samples of a uniform thickness. It is likely that the spectral processing techniques will have to be optimized with every data set acquired at different sample thicknesses, and possibly for data acquired with different spectrometers. For *in vivo* assessments, it is conceivable that a NIR method where water content relative to the amount of matrix present would be evaluated instead of absolute water content could be more useful.

In conclusion, we have developed a method to collect NIR spectral imaging data from dehydrating cortical bone samples in an environmentally-controlled chamber, which allowed for consistent measurements of bone water, and correlation of gravimetric and spectral data by PLS analysis to generate a water calibration curve. Further, future studies can continue to develop NIR water and matrix absorbances as potential biomarkers to provide insight into cortical bone quality by elucidation of the role of changes in water binding to collagen. Together NIRSI and appropriate analyses can shed light into primary molecular differences that can provide a foundation for development of techniques to assess compositional changes in bone with aging, disease states, and therapeutics.

## Methods

### Tissues

The environmental chambers were validated using NIRSI data obtained from tibiae from young bovine bone (Research 87, Bolyston MA). The calibration curve data were obtained from cadaver human tibiae harvested from 19 donors with no evidence of skeletal disease (13 male and 7 females, exempt from IRB protocols) (NDRI, Philadelphia, PA). Bones were stored frozen at −20 °C and thawed for gravimetric and NIRSI data acquisition.

### Bone sample preparation

Bovine and human cadaveric cortical bone samples were prepared for data collection as follows: Tissues were cut cross-sectionally to a uniform thickness of 500 μm from the regions of maximum cortical bone thickness (~10% distance proximal to distal endplate) with a diamond wafering saw (Buehler Isomet 1000, Lake Bluff, IL). The samples were ultrasonicated (60 khz FS60D Fisher Scientific) in 1% tergazyme solution for 2 hours at 38 °C to remove bone marrow. Marrow-free specimens were stored in phosphate buffered saline-protease inhibitor (PBS-PI) (PBS 1X, pH 7.4, Invitrogen, Carlsbad, CA) with, protease inhibitor (Sigma-Aldrich, St. Louis, MO), at −20 °C until data collection.

### Environmental chamber design

#### Small chamber

To evaluate bone samples under constant humidity, an initial chamber was constructed from a glass microscope slide base, laser cut cast acrylic walls and lid, and a glass cover slip viewing window (Fig. 1). Plastic inlets and outlets were installed on the top of the chamber to facilitate airflow. An Arduino driven Bosch BME-280 humidity sensor (Stuttgart, Germany) logged RH data inside the chamber. Humidified air flow was generated from a 4 psi pump aerating a 50 mL conical vial partly filled with room temperature water and fed into the imaging chamber through plastic tubing. This setup maintained a 72 ± 2% RH environment inside the chamber for over two hours. A desiccated environment was achieved by feeding the chamber with compressed air at 4 psi.

#### Large chamber

To collect NIRSI data from larger, clinically-relevant human bone samples, a larger imaging chamber was constructed from cast acrylic and two 76.2 × 76.2 × 0.5 mm Corning® Gorilla® Glass imaging windows (Corning, NY) (Fig. 2). Like the previous chamber model, RH data was logged from an Arduino-driven Bosch BME-280 humidity sensor. When compressed air at 4 psi entered the large imaging chamber, the internal RH quickly dropped to 0% and that level was maintained for the experiment duration. This insured no environmental moisture was absorbed by the bone samples.

### Environmental chamber validation

NIRSI data were collected from a specified 400 × 400-micron region from bovine bone samples inside the environmental chamber using a Perkin Elmer Spotlight 400 imaging system equipped with a mercury cadmium telluride (MCT) detector (Shelton, CT) every 15 minutes over the course of one hour before, and again after, a 24-hour lyophilization in a lyophilizer (Martin Christ-Alpha 1–2) (Figs 3 and 4). Chambers were validated by the assessment of changes in the 5184 cm^−1^ absorbance within a 60-minute timespan. The 24-hour lyophilization period was chosen after confirmation in pilot studies that no additional water was lost after this period of lyophilization. The samples before and after lyophilization were termed wet and dry bone, respectively.

#### NIRSI data collection for chamber validation

The spectral data were collected at 50 μm spatial resolution with 32 coadded scans at 64 cm^−1^ spectral resolution in the frequency range from 4000–7800 cm^−1^. The experimental setup depicted in Fig. 2 allowed the small chamber to maintain RHs of less than 3% and at 0%. The small chamber was additionally tested at high RH of ~70%.

#### NIRSI data processing and analysis for chamber validation

The spectral data collected at low and high RH were quantified for bone water content by processing raw data to derive the second derivative (SavitzkyGolay, 2^nd^ order polynomial and 7 points of smoothing, inverted to make peaks positive) average pixel intensity, and assessment of an established NIR water absorbance of 5184 cm^−1^ in ISys 5.0 software (Malvern Instruments, UK). It was previously shown that the average pixel intensity measurement at 5184 cm^−1^ generally correlates to the water present in the sample^20^.

Mean values ± standard deviations are reported for quantitative data. Analysis of Variance (ANOVA) with Tukey post hoc test was used to evaluate the differences in mean values for experimental data, with p < 0.05 considered statistically significant.

### Water calibration curve experimental setup

**Figure 8:**
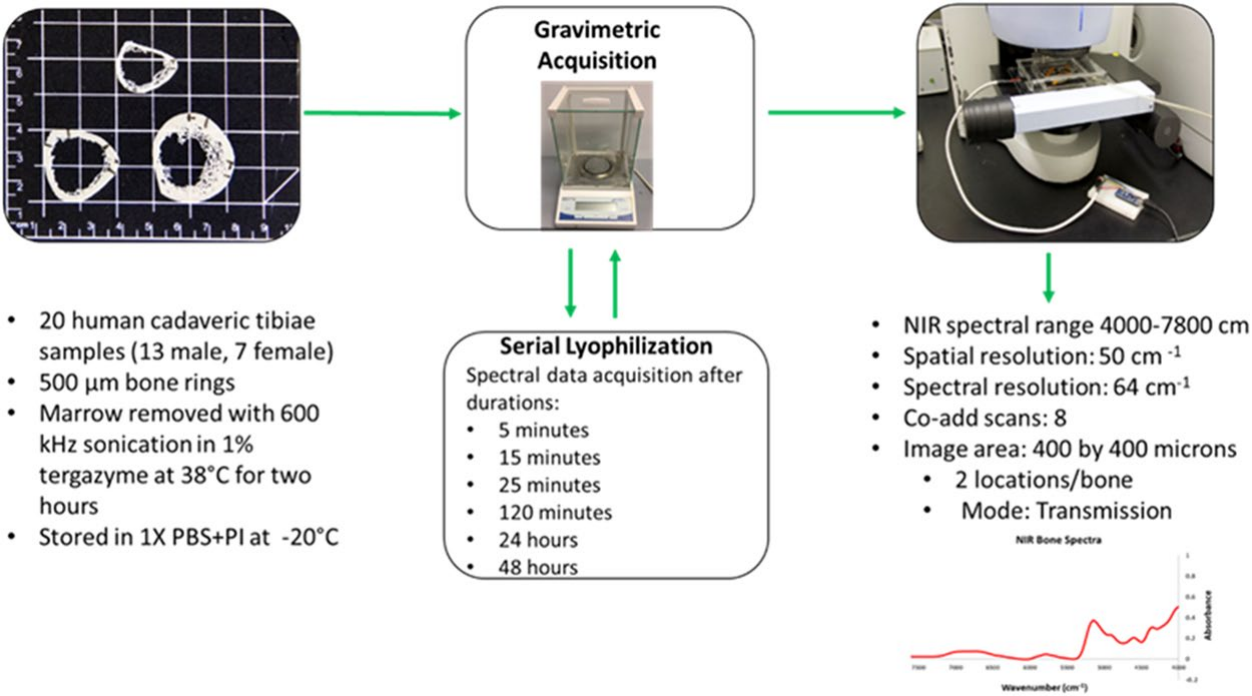
Schematic of the experimental setup for data collection from human cadaveric tissue samples used in creation of the water calibration curve.

The optimal conditions determined in the chamber calibration process were applied here to generate the water calibration curve (Fig. 8). The large chamber was used to collect NIRSI data at 0% RH for the water calibration curve. The experiment consisted of sequential dehydration of human cortical bone tissues through lyophilization and collection of NIRSI data at specific timepoints. Gravimetric and NIRSI data were collected from two different locations in each specimen at the following 7 timepoints: lyophilization for 5 minutes to uniformly remove excess surface water, then subsequent serial lyophilization for 15, 25, and 120 minutes, and 24 and 48 hours.

### Gravimetric data collection and analysis for calibration curve

Initially, human cortical bone samples were thawed and dabbed dry with a Kimwipe to remove residual PBS-PI solution. Samples were weighed after every lyophilization treatment on a SI 215D Denver Instruments (Bohemia, NY) Precision Balance for gravimetric analysis. To determine the percent of water content within the tissues the wet weights were calculated as follows:

Equation 1:

$$\frac{{Wet}\,{weigh}{{t}}_{({W})}\,-{dry}\,{weigh}{{t}}_{({D})}}{{Wet}\,{weigh}\,{{t}}_{({D})}}\,\ast \,{\rm{100}}$$

- Wet weight_(w)_ = sample weight at every dehydration timepoint.
- Dry weight_(D)_ = sample weight with 48 hours of lyophilization.

### NIRSI data collection for calibration curve

NIR spectral data was collected from two different 400 × 400-micron marked areas for each sample using a Perkin Elmer Spotlight 400 imaging spectrometer. The NIRSI data was collected in the large environmentally controlled chamber with a RH of <3% at a frequency range 4000–7800 cm^−1^ at 64 cm^−1^ spectral resolution and 50 μm spatial resolution with 32 coadded scans, and an approximate imaging time of 2 minutes at each location.

### NIRSI data processing and analysis for calibration curve

NIR spectral image were analyzed using ISys 5.0 and UnscramblerX 10.4 (Camo,Norway) software. One average spectrum was calculated for each spectral image. For 19 human cadaveric samples, a total of 302 averaged spectra were used for data processing. Second derivative processing (Savitzky Golay, 2^nd^ order polynomial and 7 points of smoothing) was applied to normalize and resolve broad peaks in NIR spectra. The optimum number of 7 smoothing points was chosen after careful analysis of spectra, since the goal was to minimize noise while maximizing peak resolution in the dataset (see Supplementary Fig. S2). NIR second derivative absorbances (inverted, to make peaks positive to facilitate understanding data) were evaluated from water components at 5184 cm^−1^, 6560 cm^−1^ and 7008 cm^−1^ and from matrix at 4608 cm^−1^ and 6688 cm^−1^ ^20^. Approximately 82 data points were removed from the analysis either due to spectral artifacts caused by residual fat in tissues or to poor quality spectra based on low signal to noise. Therefore, 220 spectra were utilized in total for both univariate and multivariate analysis.

#### Univariate analysis

Inverted second derivative spectra at 5184 and 7008 water absorbances were correlated to gravimetrically-derived water content (see Supplementary Table S1). The correlation R, p-values and RMSE are reported in Supplementary Table S1.

#### Multiple linear regression (MLR)

MLR is a statistical technique that helps to predict outcome variables based on one or more explanatory variables. MLR helps to model the linear relationship between the explanatory and outcome variables. In this study, the outcome variable is the gravimetric water content and the explanatory variables are the individual frequencies of water (5184 cm^−1^ and 7008 cm^−1^) and matrix (4608 cm^−1^ and 6688 cm^−1^). The strength of the regression is determined by high R and low RMSE values.

#### Multivariate partial least squares analysis

Partial least squares (PLS) analysis is a statistical method used to find linear relationships between predictors and determinant variables^52^ (Fig. 6 and see Supplementary Table 2). The main advantage of using PLS in creating a linear regression is the ability to analyze multiple variables at a single time. This is very useful for analysis of NIR spectra as the spectra are typically composed of overlapping absorbance bands. PLS models were developed to predict percent water content with X determinants as the spectral data and Y predictors as the gravimetrically-determined percent wet weight. Spectra were randomly chosen (N = 147) to build the model using a leave one out cross validation technique and the remaining 73 spectra were used for independent prediction. This process was repeated 3 times with spectra randomly chosen each time for model building and independent prediction. The strength of the models generated, and the independent predictions were determined based on a high R^2^ and low root mean square error of cross validation (RMSECV) values. The two main outputs in PLS models are the scores and loading plots. The scores plot visually explains the properties of samples by separating the data based on similarities and differences within the spectra. The loadings (or factors) plot explains the variance by examination of specific frequencies that cause the separation in the data structure of the scores plot. Most of the variance in data is explained by the first few factors.

## Supplementary information

Dataset 1
