# Supplementary material for: Environmentally-Controlled Near Infrared Spectroscopic Imaging of Bone Water
Source: Sci Rep. 2019 Jul 15;9:10199. doi: 10.1038/s41598-019-45897-3 (PMC6629628; doi:10.1038/s41598-019-45897-3)
Supplement: Supplementary file 1 — Dataset 1 [file 41598_2019_45897_MOESM1_ESM.pdf]

Environmentally-Controlled Near Infrared Spectroscopic Imaging of Bone Water

Ramyasri Ailavajhala[1], Jack Oswald[1], Chamith S. Rajapakse[2], Nancy Pleshko[1*]

1. Department of Bioengineering, Temple University
2. Departments of Radiology and Orthopaedic Surgery, University of Pennsylvania

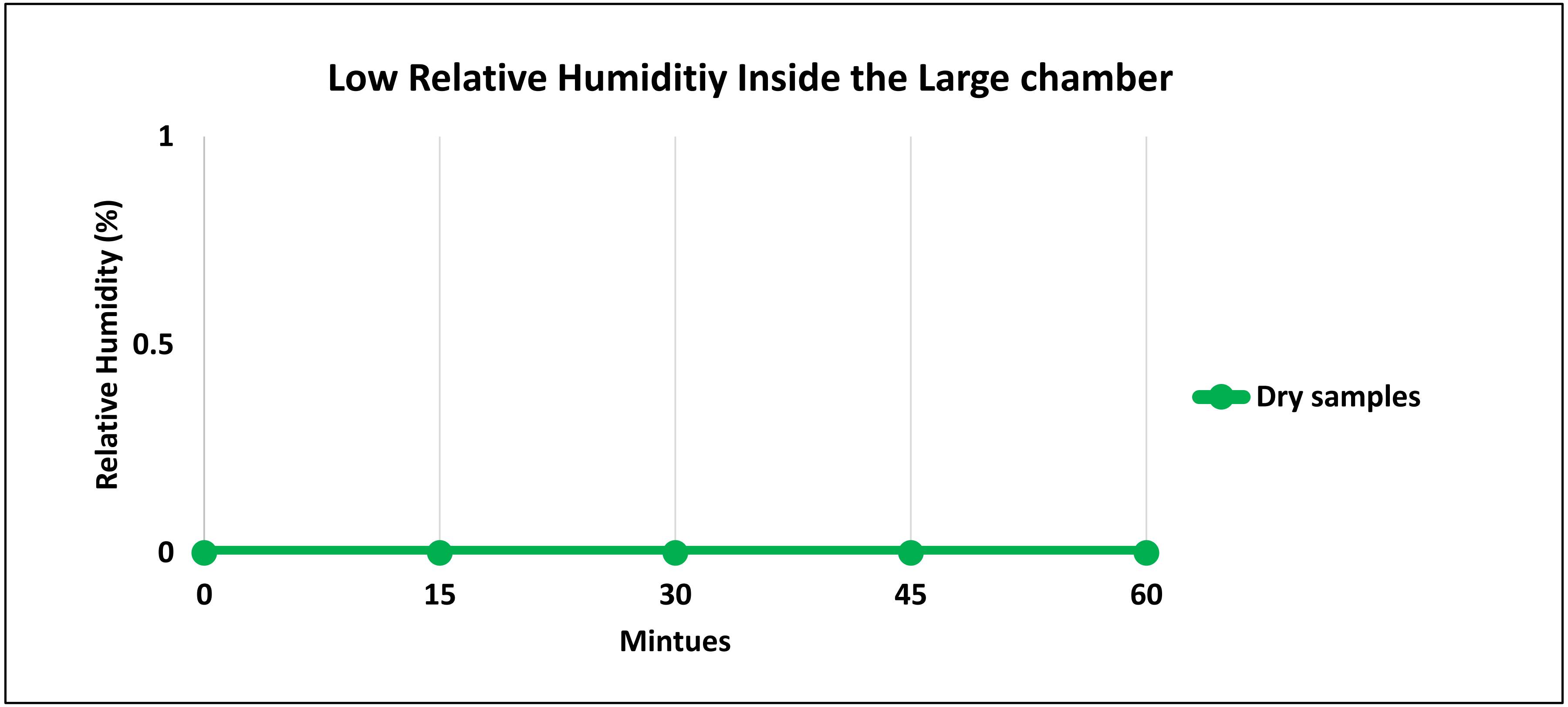

Supplementary Figure S1: Relative humidity inside the large imaging chamber during one hour of spectral data collection of dry bone samples in low RH conditions.

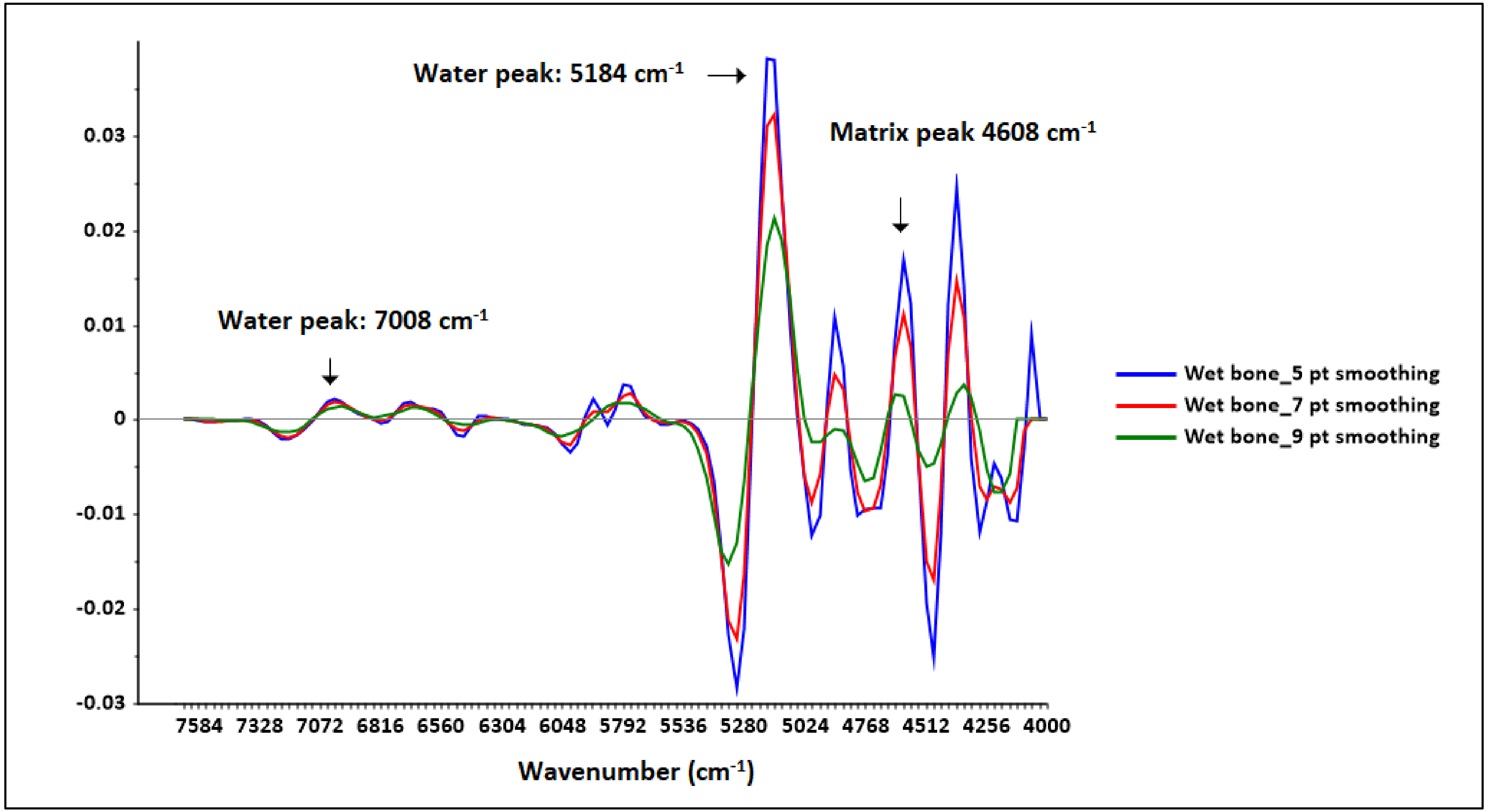

Supplementary Figure S2: Inverted second derivative spectra of wet bone spectra with varying smoothing points.

Table 1: Univariate Analysis

Frequencies	Component	R-value	RMSE
5184 cm^{-1}	Water	0.84, $p<0.05$	12.50%
7008 cm^{-1}	Water	0.63, $p<0.05$	16.8%
5184/4608	Water/Matrix	0.96, $p<0.05$	9.90%
7008/6688	Water/Matrix	0.90, $p<0.05$	18.6%
5184 cm^{-1} + 4608 cm^{-1}	Water +Matrix	0.95, $p<0.05$	8.90%
7008 cm^{-1} + 6688 cm^{-1}	Water + Matrix	0.93, $p<0.05$	9.30%

Table 2: Multivariate Analysis: Results from the PLS best model

R-value	RMSECV
0.92, $p<0.05$	6.60%
